# ‘How low can you go?’ Developers’ perspectives on involving young children in the development of patient reported outcome measures

**DOI:** 10.1186/s41687-025-00924-y

**Published:** 2025-07-15

**Authors:** Victoria Gale, Philip A. Powell, Jill Carlton

**Affiliations:** 1 Leeds Beckett University, Leeds, UK; 2 School of Medicine and Population Health, University of Sheffield, Sheffield, UK

**Keywords:** Concept elicitation, Cognitive interview, Children, Patient reported outcome measures, Qualitative research

## Abstract

**Background:**

Recommendations suggest that children need to be ≥ 8 years-old to participate in concept elicitation (CE) and cognitive interviewing (CI) when developing patient reported outcome measures (PROMs). However, these recommendations have not been subject to thorough scrutiny and recent evidence suggests that younger children may be enabled to participate. This study audited current opinions of PROM developers regarding the feasibility of conducting CE and CI research with children.

**Methodology:**

An online survey was developed to capture PROM developers’ perspectives, recruited from existing networks (UK PROMs, International Society for Quality of Life Research) and outcomes research groups from English-speaking countries between August-November 2024. Survey questions explored the ages from which developers considered it feasible to include children in CE and CI research, their previous experiences conducting CE/CI research with children, and respondents’ background experiences with children. Results were analysed descriptively, and exploratory comparisons were made based on developers’ characteristics.

**Results:**

Fifty-eight responses were analysed. The mean youngest ages considered feasible to include children in CE and CI research were 6.66 years and 7.36 years, respectively. The mean youngest ages respondents reported involving children in CE and CI research in practice were 7.67 years and 8.13 years, respectively. Concern that children would have insufficient cognitive and/or linguistic skills was the most often endorsed reason for considering the involvement of younger children to be infeasible. Respondents who had recent parental experience with younger children tended to consider it feasible to include children from younger ages. Those who had conducted CI with children considered it feasible to include children in CI from younger ages. Opposingly, those who had conducted CE with children considered it less feasible to include younger children in CE research.

**Conclusions:**

In-line with established precedent, PROM developers included children from ∼ 8 years-old in CE and CI research, while in principle considering it feasible to include younger ages. Reasons for including (or not including) certain age groups in CE and CI research need critical evaluation and PROM developers may wish to consider ways in which more inclusive opportunities for younger children can be provided.

**Supplementary Information:**

The online version contains supplementary material available at 10.1186/s41687-025-00924-y.

## Background

During patient reported outcome measure (PROM) development and evaluation, evidence for instrument content validity (i.e., relevance, comprehensiveness, and comprehensibility for the intended target population) is required [1–3]. Qualitative research, including concept elicitation (CE) and cognitive interviewing (CI), is widely used and recommended to evidence content validity [4–7]. CE interviews and/or focus groups are used to investigate the target population’s experiences of a health condition to help inform the development of the PROM’s conceptual framework and content in a way that is grounded in the target population’s lived experience [6–8]. CI specifically aims to evaluate whether the target population understands PROM content as intended, and whether that content is relevant and comprehensive for them [2, 4]. Children, as well as adults, should be involved in CE and CI wherever possible when they are the intended target population for the PROM [9, 10]. The earlier stage of development of *young* children (i.e., < 8 years) means including this age group is particularly important; it cannot be assumed that they will interpret instruments consistently with the way intended by *adult* developers [9, 11, 12].

The age from which children can typically participate (see Table 1 for study definitions) in CE and CI research is ambiguous, with the feasibility of including young children aged ≤ 7 years being particularly unclear [13]. For example, young children may be easily led by social desirability bias [14, 15], may be intimidated or anxious about the novelty of the interview or focus group situation [16], and CI in particular may require abstract thinking skills that younger children have not yet developed [9]. Within the published literature there are limited examples of children aged ≤ 7 years participating in qualitative PROM development/evaluation [13]. Further, there is little guidance available for researchers as to how *young* children can be involved in CE and CI; existing recommendations focus primarily on children aged ≥ 8 years, and it is generally suggested that PROM developers can only be confident that children can participate in qualitative PROM development/evaluation from 8-years-old [9, 10].

Existing recommendations [9, 10], however, are now more than 10 years old and have not been subject to thorough empirical scrutiny. Further, recent evidence suggests that younger children (5–7 years) may have the necessary skills (i.e., cognitive, linguistic, and social) to be supported to participate in CE and CI research (e.g [17, 18]). As such, it is possible that other factors are contributing to the lack of involvement of younger children in qualitative aspects of PROM development/evaluation. When considering who to involve in qualitative development/evaluation research activities PROM developers will likely be influenced by: (1) existing guidance and/or regulations they choose or are required to follow (which, as discussed, are typically sceptical of young children’s abilities to participate in CE and CI); and (2) their own previous experiences and opinions [19].

Historically children were excluded from health and social research because of perceptions that they were incapable and unreliable research participants [20, 21]. Researcher’s own opinions regarding children’s competence can influence the age from which children are included in research [22]. It is therefore possible that young children are not being routinely involved in CE and CI because developers do not *believe* this age group can participate meaningfully (using standard methods) and thus are not attempting to involve them in research (e.g., by adapting methods and developing solutions). Alternatively, PROM developers may lack confidence conducting CE/CI research with young children; recommendations often emphasise the skill needed by researchers to be able to effectively involve young children in qualitative research (e.g., adapting to children’s language and maintaining children’s attention) [9, 10].

Practical constraints may also be limiting the ability of PROM developers to include younger age groups in CE and CI projects. For example, there are few recommendations available for including young children specifically in CE/CI research [13, 23]. Navigating gatekeepers (e.g., parents/guardians, professional children’s organisations such as schools) can be challenging and may limit the feasibility of recruiting young children [22, 24]. Obtaining ethical approval for research with young children may also be challenging, particularly given the additional ethical considerations associated with including younger age groups, such as how informed consent or assent can be obtained and how power imbalances between researchers and children can be reduced [22, 24, 25].

To further develop knowledge of *if* and *how* young children can meaningfully participate in qualitative aspects of PROM development/evaluation there needs to be greater discussion between PROM developers [24, 26]. A first step towards this process would be to know what perceptions PROM developers have, what they do (or do not do) when creating PROMs for children, and what is contributing to these decisions. As such, this study aimed to audit via an online survey the current opinions of PROM developers regarding the feasibility of including children in qualitative aspects of PROM development/evaluation. There were four research questions:

1. From what age is it considered feasible for children to participate in CE and CI as part of PROM development/evaluation?
2. What perceived challenges/factors are limiting the feasibility of children’s participation in CE and CI?
3. What evidence or information would be needed to demonstrate the feasibility of children’s participation in CE and CI?
4. Are self-report PROMs being developed for children where the intended target age range of the instrument is wider than the age range of children included in CE and CI?

In addition, exploratory comparisons were conducted to investigate potential differences of PROM developers’ opinions/perceptions based on key characteristics (i.e., parental experience, professional/voluntary experience with children aged ≤ 11 years, and previous experience with CE and CI projects with children).

**Table 1:** Definitions of key terms

Key term	Definition
PROM developer	Having been listed as an author on **at least one** published PROM development* paper **AND** having participated in **at least one** of the following activities: • Development of a conceptual model (e.g., concept elicitation) • Item development • Cognitive interviewing/debriefing to pre-test items • Item reduction/selection • Psychometric testing *this **CAN** include the development of a descriptive system for a preference-based measure (PBM), sometimes known as a preference-weighted measure (PWM)
Children	People aged 0–18 years
Participation	The child independently: 1. Engages with the data collection task (e.g., listening to the interviewer, following task instructions) **AND** 2. Thinks about the interview questions/prompts (e.g., thinking about symptoms of a health condition) **AND** 3. Communicates their own views and ideas (e.g., verbally or non-verbally expressing their thoughts, drawing pictures about a health condition if asked to by the interviewer) Adults (e.g., parents) may be expected to support their child provided they do not influence the child’s answers e.g., supporting the child to read or gently re-directing the child back to the interview task. It would *not* include answering questions on behalf of their child or leading the child’s thinking or communication of ideas.
Feasibility	Possible to collect meaningful data from participants that is valuable to the aims of the research. With children, this may include the use of age-appropriate methods of data collection (e.g., shorter interview times, appropriate vocabulary for questions/prompts, potentially using props or creative activities).
Concept elicitation	Qualitative research (e.g., interviews/focus groups) where representatives of the target population are asked about their experiences of the health condition. The information gathered is used to develop a conceptual framework and to help identify the health concepts to be included in the PROM and the generation of PROM content.
Cognitive interviewing (sometimes known as “cognitive debriefing”)	Qualitative interviews conducted with representatives of the target population which aim to evaluate the content validity (comprehensiveness, comprehensibility, relevance) of the draft PROM. Typically, the participant is asked to complete the draft PROM while also being asked direct verbal probes and/or to ‘Think Aloud’ such that the interviewer gains an insight into their thought processes. This information is used to evaluate the PROM’s content validity.

*Abbreviations*: PROM (patient reported outcome measure); PBM (preference−based measure); PWM (preference−weighted measure)

## Methods

### Study design

An exploratory, observational survey study was conducted to descriptively audit a snapshot of PROM developers’ opinions regarding the involvement of children in qualitative PROM development/evaluation research. Quantitative data were collected via an online survey hosted on Qualtrics. Ethics approval for the study was granted by the School of Medicine and Population Health Research Ethics Committee at the University of Sheffield (reference number: 063895). 

### Participants and recruitment

Eligible participants were PROM developers able to complete an online survey in English identified through self-report screening questions presented at the start of the survey; if the respondent stated that they did not meet either of the two criteria (Table 1), the survey thanked them for their interest and closed.

Recruitment involved opportunity and snowball strategies. Existing networks and research groups of PROM developers from English-speaking countries (UK, US, Canada, New Zealand, and Australia) were contacted via known or publicly available contacts and asked if they would be willing to circulate an invitation to participate through their networks. Networks included the International Society for Quality of Life Research (ISOQOL), the UK PROMs Network, and outcomes research groups at different universities, including Universities of Leeds (UK), Melbourne (Australia), and Duke University (US). A full list of networks and groups approached is included in Supplementary File 1. Additionally, links to the survey were distributed in-person at the ISOQOL conference in October 2024.

The study did not aim to confirm pre-specified hypotheses, and null hypothesis significance testing was not planned, meaning power analysis was inappropriate. As such, given the exploratory nature of the study, sample size was determined by the maximum number of eligible participants it was possible to recruit during the time the survey was open (August-November 2024).

### Survey

**Fig. 1:**
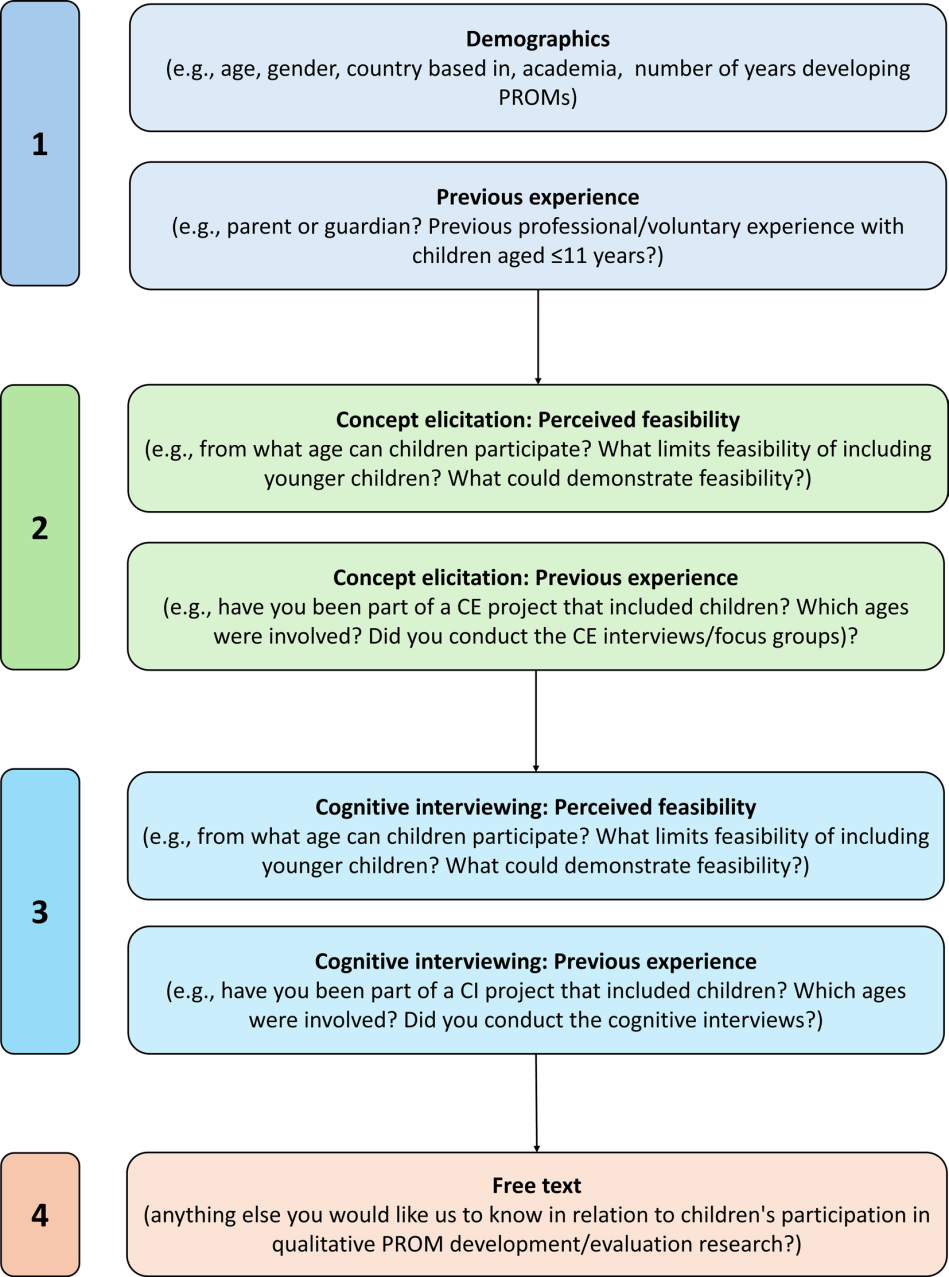
Overview of survey content. All survey questions are included in Supplementary File 2

The full list of survey questions is included in Supplementary File 2. The survey was formed of four main sections (Fig. 1): (1) respondent demographics and experience with children; (2) perceived age from which CE research is typically feasible and previous experience conducting CE research with children; (3) perceived age from which CI research is typically feasible and previous experience conducting CI research with children; and (4) free-text option to share any additional information. Aside from the final free text question all survey questions had multiple choice response formats. Clear definitions of key terms (Table 1) were provided throughout the survey, including via pop-up text when respondents clicked on key terms.

The survey was pilot tested to check for usability and clarity by two researchers from the UK and Australia with experience developing PROMs and measuring children’s health-related quality of life (HRQoL). They did not then participate in the survey. Minor modifications to the layout and formatting were made following the pilot test, but no changes were made to the survey content.

### Analysis

Data were analysed descriptively (i.e., calculation of frequencies and percentages), auditing PROM developers’ perspectives. Exploratory descriptive comparisons of perceived feasibility were made based on: (1) parental status (overall and to a child ≤ 11 years); (2) professional/voluntary experience with children ≤ 11 years; (3) part of a CE or CI project with children (≤ 7 years or ≥ 8 years); and (iv) direct interviewing experience with children. Qualitative data in free text comments were thematically synthesised. Key themes in free text comments were identified for each survey question and were compared descriptively across questions for similarities and differences.

## Results

The full anonymised dataset supporting this study is provided in Supplementary File 3.

### Sample characteristics

A total of 58 responses were included in the analysis. Out of 65 eligible responses (i.e., the respondent was a PROM developer and had provided consent), six were excluded for being incomplete and one was excluded because it was unclear whether the respondent had interpreted the survey questions as intended (i.e., they reported conducting CE/CI with children from 1-year-old independently). Most respondents were female (67%), had five or more years’ experience developing PROMs (82.8%), and had previous experience working with children aged ≤ 11 years in a professional or voluntary capacity (77.6%) (Table 2). Fifty percent of respondents were primarily based in academia and 44.8% were based in the UK.

**Table 2:** Participant demographics

**Gender**:	*N* (%)
Male	19 (33%)
Female	39 (67%)
Age (years)	46 (13.73)
**Primary setting**:	
Academia	29 (50%)
Industry	20 (34.5%)
Other	9 (15.5%)
**Country**:	
UK	26 (44.8%)
US	14 (24.1%)
Canada	7 (12.1%)
Australia	2 (3.4%)
Europe (not UK)	9 (15.3%)
**Years of experience developing PROMs**:	
0–1 year	1 (1.7%)
2–4 years	9 (15.5%)
5 + years	48 (82.8%)
**Parent or guardian**	38 (65.5%)
Parent or guardian to a child/children aged ≤ 11 years	16 (27.6%)
Experience working* with children aged ≤ 11 years	45 (77.6%)

*Participants were asked if they had “ever worked with children aged 11 years and younger in a professional or voluntary capacity (e.g., researcher, teacher, healthcare professional, childcare worker, children’s activities volunteer etc.)”

### Perceived feasibility of PROM development

#### Minimum ages

**Fig. 2:**
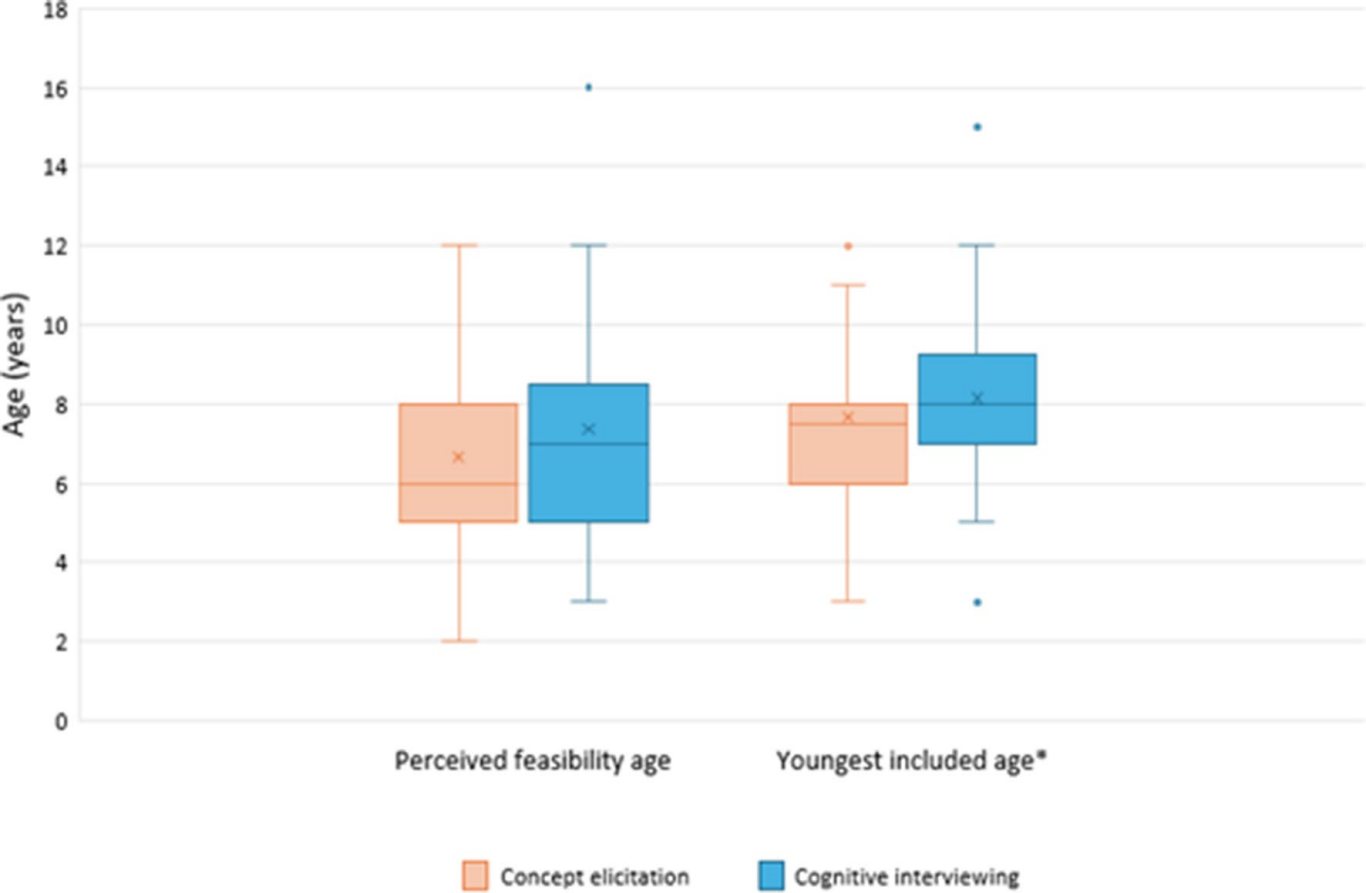
Youngest ages respondents perceived it was feasible to include children in concept elicitation and cognitive interviews, and youngest ages respondents included in concept elicitation and cognitive interviews in practice. Graph shows median youngest ages (solid line), mean youngest ages (“X”), and interquartile range (box)

The mean minimum ages respondents believed it feasible to involve children in CE and CI were 6.66 years (SD = 2.29, range = 2–12 years) and 7.36 years (SD = 2.65, range = 3–16 years) respectively (Fig. 2)^1^. In practice, the mean youngest ages respondents reported having involved children in CE and CI were 7.67 years (SD = 2.28, range = 3–12 years, *n* = 36) and 8.13 years (SD = 2.44, range = 3–15 years, *n* = 38).

#### Reasons for perceived infeasibility in involving younger children

**Fig. 3:**
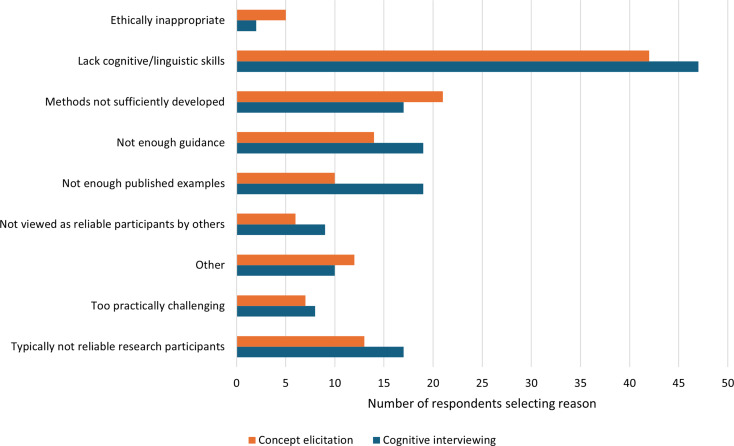
Reported reasons why conducting concept elicitation/cognitive interviewing research with children younger than the reported feasibility age would not be typically feasible (there were no restrictions on the number of reasons that could be selected)

Having reported the age from which they believed it typically feasible for children to participate in CE and CI, respondents were asked why they thought it would be infeasible for *younger* children to participate. Shown in Fig. 3, the most common reason selected was that younger children would “not typically have the cognitive and/or linguistic skills needed” to participate in the research (*n* = 42, (32%) for CE; *n* = 47 (33%) for CI). Not having “enough published examples” was notably endorsed more often for CI (*n* = 19, 13.48%) compared to CE (*n* = 10, 7.69%).

“Other” reasons reported in free text comments further elaborated on younger children not having sufficient skills (e.g., *“It depends on their reading level and also their energy/attention”)*, but also acknowledged variability in skill levels among young children (e.g., *“Some children have the skills needed to participate*,* but since the question states ‘typically’ then as a whole group*,* children in this younger age group would not consistently have those skills needed”)*.

#### Evidence needed to demonstrate feasibility

**Fig. 4:**
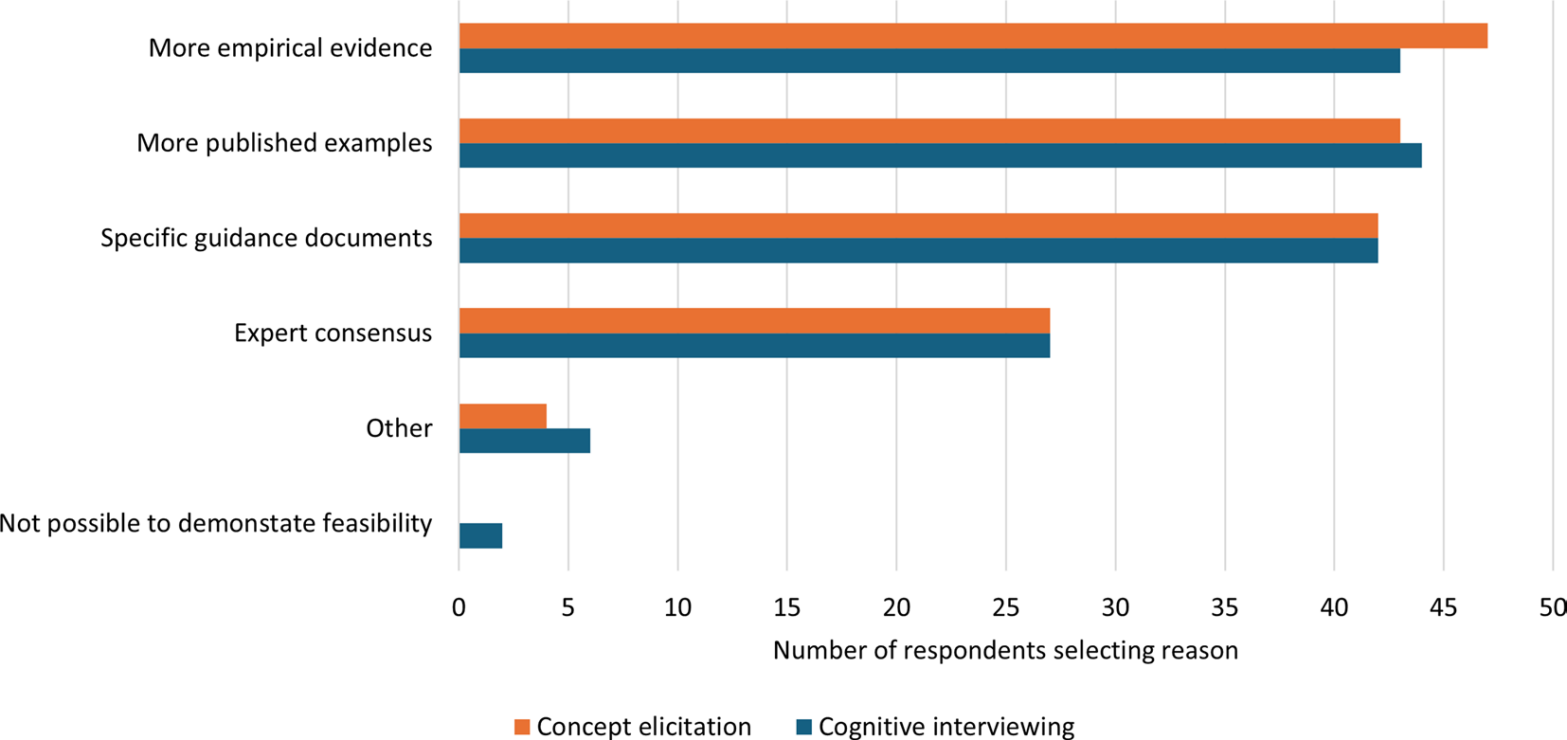
Number of respondents reporting factors that would be needed to demonstrate the feasibility of children younger than currently perceived feasible participating in concept elicitation and cognitive interviewing research (there were no restrictions on the number of response options that could be selected)

Having more empirical evidence, more published examples, and more specific guidance documents were all highly endorsed as necessary to convince respondents of the feasibility of including younger children in CE and CI (Fig. 4). Only for CI did a minority of respondents report that it would not be possible to demonstrate the feasibility of younger children participating (*n* = 2, 1.25%). The youngest ages these respondents believed it feasible to include children in CI were 7 and 10 years.

In free text responses it was reported that published evidence, research and guidance would need to represent “*a wide range of background/social characteristics*” and one respondent reported that “*personal experience*” would be needed to demonstrate the feasibility of involving young children in CE and CI.

#### Missed opportunities to involve young children

Ten respondents reported that they had been part of a CI project where the youngest children a self-report PROM was intended for had not been involved in CI. Half as many (*n* = 5) reported this was true for CE research. The most common reason selected for these ‘missed opportunities’ was that the data collection task was considered too complex for young children and so they were not invited to participate.

### Exploratory descriptive comparisons

#### Concept elicitation

**Fig. 5:**
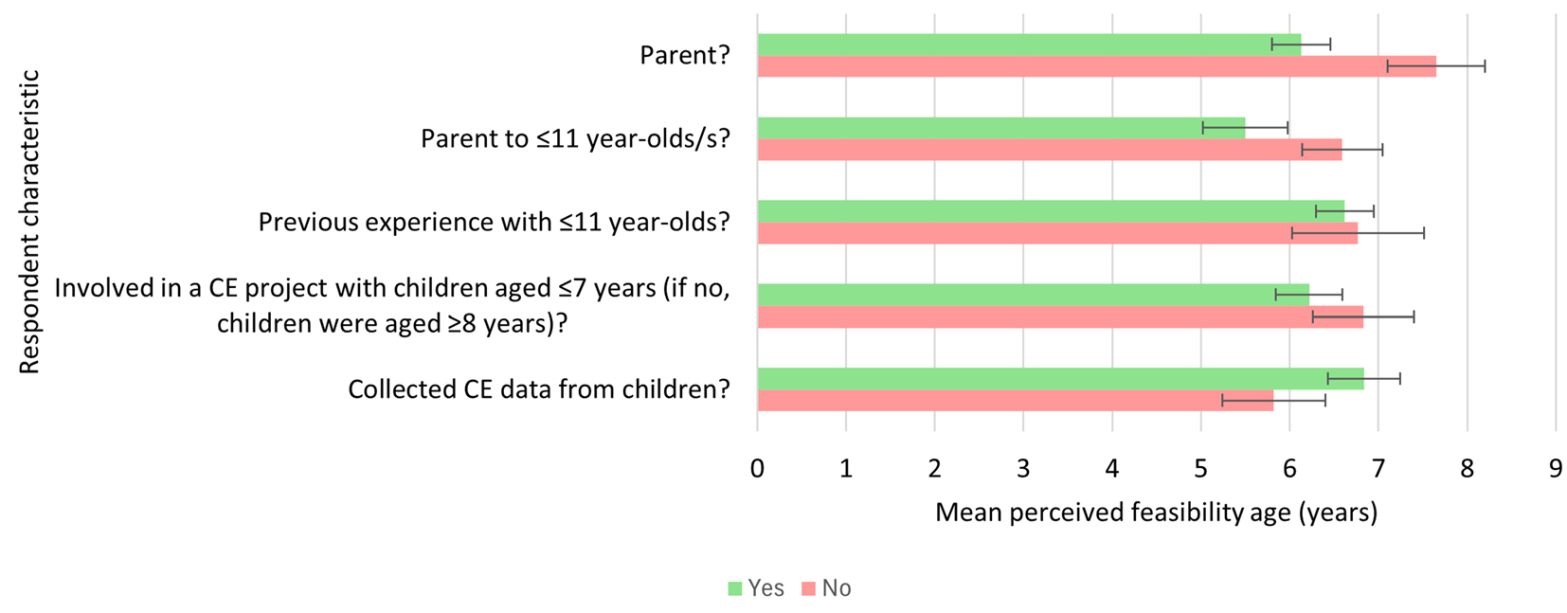
Comparison of mean perceived feasibility ages for concept elicitation research across respondents with different background characteristics. Green bars show mean perceived feasibility ages for respondents with the characteristic, red bars show mean perceived feasibility ages for respondents without the characteristic. Error bars show standard error of the mean. CE = concept elicitation

The mean perceived age from which CE was considered feasible was lower for respondents who were parents at 6.13 years (SD = 2.04, range = 2–11 years, *n* = 38) compared to those who were not at 7.65 years (SD = 2.46, range = 4–12 years, *n* = 20) (Fig. 5). Similarly, the mean age was lower for those who were parents to children aged ≤ 11 years at 5.5 years (SD = 1.79, range = 2–8 years, *n* = 16) compared to those who were parents to children aged ≥ 12 years at 6.59 years (SD = 2.13, range = 4–11 years, *n* = 22). Mean ages were higher for respondents who had directly collected data from children in a CE project at 6.84 years (SD = 2.04, range = 4–12 years, *n* = 25) compared to those who had been involved in a CE project with children but had not been responsible for data collection (5.82 years, SD = 1.94, range = 4–11 years, *n* = 11).

The mean perceived ages from which CE was considered feasible were comparable across respondents who had previous professional or voluntary experience with children aged ≤ 11 years and those who did not. For respondents who had been part of a CE project with children (*n* = 36), mean ages were comparable for those who had involved children from age ≤ 7 years and those who had involved children from age ≥ 8 years.

#### Cognitive interviewing

**Fig. 6:**
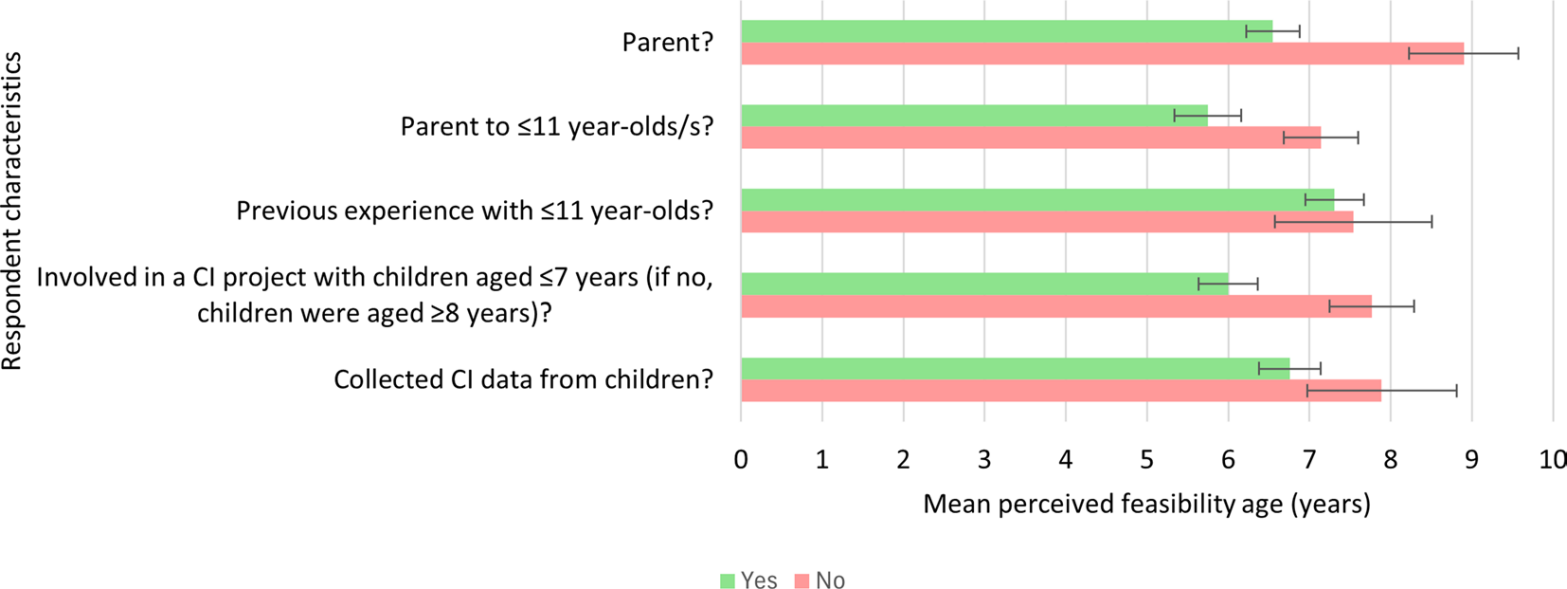
Comparison of mean perceived feasibility ages for cognitive interviewing across respondents with different background characteristics. Green bars show mean perceived feasibility ages for respondents with the characteristic, red bars show mean perceived feasibility ages for respondents without the characteristic. Error bars show standard error of the mean. CI = cognitive interviewing

As with concept elicitation, the mean perceived age from which CI was considered feasible was lower for respondents who were parents at 6.55 years (SD = 2.05, range = 3–12 years, *n* = 38) compared to those who were not at 8.9 years (SD = 3.01, range = 4–16 years, *n* = 20) (Fig. 6). Similarly, the mean age was lower for those who were parents to children aged ≤ 11 years at 5.75 years (SD = 1.65, range = 3–8 years, *n* = 16) compared to those who were parents to children aged ≥ 12 years at 7.14 years (SD = 2.15, range = 4–12 years, *n* = 22).

Differently from concept elicitation research, the mean age from which CI was considered feasible was also lower for respondents who had been part of a CI project with children from age ≤ 7 years at 6 years (SD = 1.46, range = 4–10 years, *n* = 16) compared to those who had been part of a CI project with children from age ≥ 8 years at 7.77 years (SD = 2.45, range = 4–12 years, *n* = 22). Mean ages were lower for those who had conducted CIs with children as part of these projects at 6.76 years (SD = 2.05, range = 4–12 years, *n* = 29) compared to those who had not at 7.89 years (SD = 2.76, range = 5–12 years, *n* = 9). Mean ages were comparable across participants who had previous professional or voluntary experience with children aged ≤ 11 years and those who did not.

### Additional free text comments

All free text comments are included in Supplementary File 3. The usefulness of adapted activities for supporting children to participate in qualitative PROM development research was often reported in free-text comments e.g., using a “*game type system with graphics*,* audio and no/minimal text*” and “*methods and measures that utilise non-verbal stimuli and items*”. Variability in young children’s skills was also often reported in free-text responses e.g., *“I don’t think there is a specific age that can be set as a cutoff –developmentally children are so different”*, as was the need to consider the potentially biasing factor of having caregivers present during data collection with children e.g., “*You would typically want to have a guardian present at the interviews*,* which could be an influencing factor in the participant’s responses*”. Challenges of obtaining ethical approval and navigating gatekeepers to be able to recruit children were also reported e.g., *“one of the challenges with involving children in research […] is the ethics application and access to them”.* Four respondents also expressed enthusiasm and a need for children’s involvement to be explored “*systematically*”, for example:

*"The inclusion of children in the development of PROs intended to measure their experiences is important, and I’m glad you are doing research on this topic. Guidelines and best practices for including children, particularly younger ones, would be especially helpful."*

## Discussion

This study has highlighted a disparity between the average youngest ages respondents considered it feasible to include children in CE and CI research (6.66 years and 7.36 years, respectively) and the often-recommended minimum age of 8-years-old [9, 10]. This finding suggests that existing recommendations may be outdated and/or inaccurate. This younger *perceived* feasibility age may also reflect a more general (gradual) shift towards considering it possible for younger children to self-report via PROMs and thus potentially participate in instrument development/evaluation research. For example, a recent survey of clinicians found that the majority would be “very likely” or “moderately likely” to use self-reported information from children via PROMs to inform symptom management during cancer treatment from children as young as 4-years-old [27].

Although considering it feasible, in principle, to include younger children, developers reported having included children on average from ∼ 8 years of age in CE and CI projects, in-line with existing recommendations [9, 10]. It is not unexpected that developers would follow existing precedent [19], particularly given uncertainty surrounding the feasibility of involving younger age groups in this type of research [13]. Further, respondents endorsed a need for further guidance, published examples, and empirical evidence that children younger than 8 years can participate meaningfully in qualitative PROM development/evaluation research. Evidence has been gradually accumulating since the publication of prior recommendations that demonstrates that younger children (i.e., from ≤ 7 years) *can* participate in CE [28–30] and CI [17, 18, 31, 32]. This evidence together with the survey results highlight a need to review existing recommendations and for PROM developers to critically question whether 8-years-old is truly a justified lower age limit for involving children in CE and CI projects.

It is important to recognise that the existing recommendations discussed [9, 10] do not propose that age thresholds for when children can/cannot participate in qualitative PROM development/evaluation are definitive [9, 10]. As reported by survey respondents, it is emphasised that substantial variability between children of the same chronological age exists [9, 10]. As such, we do not propose that a definitive lower age limit for children’s participation in CE and CI research should be set; as with any participant group, the characteristics of the individual participants, nature of the concept of interest, and complexity of the PROM will all influence the feasibility of these research activities. Rather, it is proposed that developers *should be open* to including children aged < 8 years rather than uncritically following precedent. Young children have a right to be involved in research about matters that affect them (i.e., their health) [25, 33]; they may bring unique insights that are not known by adults and their inclusion in CE/CI is essential for the development of PROMs (designed for young children) with content validity, ultimately helping support the inclusion of young children’s voices in health care, research and policy [10, 34]. Scepticism towards young children’s capabilities as research participants should be replaced with more open and inclusive attitudes in which developers assume responsibility for making CE and CI research activities as accessible as possible [17].

Given the variability between children of the same chronological age, researchers may find it useful during recruitment to discuss the interview/focus group activity with the child’s parent/carer (or another adult who knows them well such as a teacher or healthcare professional). Researchers can discuss the expectations of what the child will need to do to take part (such as those listed in Table 1) to help determine the likelihood of the child being able to participate. These conversations can also be used to identify ways in which the research activity could be adapted to children’s individual needs, such as if the child would like to share a particular story book or toy with the interviewer to help build rapport and confidence with the interviewer, supporting an inclusive approach to young children’s participation.

The exploratory comparisons conducted to compare differences in the perceived feasible minimum age of children’s involvement were descriptive only, and it is not possible to confirm causality. However, results are suggestive that those with an apparent greater lived experience with children (i.e., through parenthood), and notably a recent lived experience with young children, think that it is likely to be more feasible to include younger children as research participants in CE and CI projects. A researcher’s own experiences are widely recognised to influence the research they undertake [35–37] and personal experience likely contributes to PROM developers’ decision-making when conducting qualitative instrument development [19]. Reflexivity and reflecting on one’s own positionality as a researcher is good practice within qualitative research (e.g [35, 36, 38, 39]) yet is not emphasised in existing recommendations for conducting qualitative CE or CI research with children [9, 10] or with adults [2, 4, 5]. As such, it may be helpful for developers to explicitly reflect on and discuss any specific skills or experience they have with young children which may provide insight into the feasibility and methods of involving children in CE and CI research.

Findings also suggest greater scepticism around the feasibility of conducting CI with younger children compared to CE; perceived feasibility ages were older for CI and there were twice as many ‘missed opportunities’ reported for younger children to be involved in CI than CE projects. It is possible that this scepticism stems from concern that CI is too abstract a task for young children [9], or perceptions that the structured nature of CI [2, 4] cannot be adapted for young children. However, recent evidence has demonstrated that children aged ≤ 7 years can participate in CI with only simple adaptations to interview procedures, such as asking children to explain item meanings to a toy [17], conducting the interview in shorter sections so as not to overwhelm children [31], and having an additional adult present to help explain cognitive interview tasks [18]. Overall, children may be able to participate in CI from a younger age than was initially thought. Respondents who had conducted CI research with children gave a lower perceived feasibility age than those who had not. Accordingly, PROM developers should be open to conducting CI with younger children in the same way they are towards CE.

Some survey respondents expressed concern in free-text comments that younger children could not participate in CI because they would not have necessary reading skills. However, young children can be enabled to self-report via a PROM without the need for self-completion (i.e., an adult can read items to the child and support them in the physical marking of a response) [27]. Concern about reading age should not be a limiting factor in children’s involvement in CI unless the PROM specifically requires the child to independently self-complete. Alternatively, CI offers an opportunity to evaluate whether self-completion is feasible for younger age groups; as has been discussed, younger children *can* participate in CI with more inclusive approaches that include modifications to interview procedures as necessary [17, 18, 31, 32].

This study is not without limitations. The small sample size makes it difficult to draw definitive conclusions from the data and this is paired with a descriptive, associational analysis only. Because of practical constraints the survey was conducted in English only and it was primarily research groups in the UK and US who shared survey information with members, meaning the sample is not fully representative of the global perspective of PROM developers. Most respondents reported that they had been involved in qualitative PROM development/evaluation projects that had included children, making findings less representative of PROM developers who do not have experience conducting research with children and who may have different perceptions of feasibility. While this study has identified multiple new insights, it was not exhaustive. Some factors that may influence the involvement of young children in CE/CI projects, such as the recency of research described by survey respondents and respondent academic discipline/industry type, were not collected.

In conclusion, interest in involving younger children in qualitative PROM development/evaluation is growing and PROM developers may consider it feasible for children younger than the recommended 8-years-old to participate in qualitative aspects of PROM development/evaluation. However, the inclusion of children aged ≤ 7 years in CE and CI research is yet to become commonplace in practice. There is concern that younger age groups lack the cognitive and/or linguistic skills necessary to participate in CE and CI, despite a gradual accumulation of evidence that this age group *can* be enabled to participate meaningfully. There is also greater scepticism surrounding CI which may not be an accurate reflection of the feasibility of CI with younger children in practice. The adoption of an open approach to involving young children that places greater responsibility on the role of the researcher to find ways of enabling young children’s participation is necessary. This will help advance evidence-based qualitative approaches to facilitate inclusivity in children’s PROM development/evaluation that are based on progress not precedent.

## Electronic supplementary material

Below is the link to the electronic supplementary material.

Supplementary Material 1

Supplementary Material 2

Supplementary Material 3

## Data Availability

The full anonymised dataset supporting this study is provided in Supplementary File 3.

## Footnotes

1 Sample sizes are different. Mean perceived feasibility ages were calculated from the sample of 58 respondents. Only 36 respondents reported having been part of a CE project that had involved children, and only 38 respondents reported having been part of a CI project that involved children.

## Abbreviations

CE: Concept elicitation
CI: Cognitive interviewing
HRQoL: Health-related quality of life
ISOQOL: International Society for Quality of Life Research
PBM: Preference-based measure
PROM: Patient reported outcome measure
PWM: Preference-weighted measure
