# Supplementary material for: ‘How low can you go?’ Developers’ perspectives on involving young children in the development of patient reported outcome measures
Source: J Patient Rep Outcomes. 2025 Jul 15;9:91. doi: 10.1186/s41687-025-00924-y (PMC12263538; doi:10.1186/s41687-025-00924-y)
Supplement: Supplementary file 1 — Supplementary Material 1 [file 41687_2025_924_MOESM1_ESM.docx]

**Supplementary File 1: Networks and research groups contacted**

**Article Title:** ‘How low can you go?’ Developers' perspectives on involving young children in the development of patient reported outcome measures

Networks and research groups that shared the survey invitation among members.

| **Network/research group** | **Country** |
| --- | --- |
| ISOQOL Mixed methods SIG | International |
| ISOQOL Developing Nation SIG |  |
| ISOQOL Response Shift SIG |  |
| ISOQOL Statistics SIG |  |
| ISOQOL Patient Engagement SIG |  |
| ISOQOL Child Health SIG |  |
| ISOQOL Industry SIG |  |
| ISOQOL Clinical Practice SIG |  |
| ISOQOL New Investigators SIG |  |
| ISOQOL Australia and New Zealand SIG |  |
| UK PROMs Network | UK |
| Patient Centred Outcomes Research - University of Leeds |  |
| Clinical Outcomes Oxford Innovation |  |
| Center for Patient-Centered Outcomes - Northwestern University | USA |
| Center for Health Measurement - Duke University |  |
| Center for Health Services and Outcomes Research - John Hopkins, Bloomberg school of public health |  |
| QUOKKA Research Program - University of Melbourne of Sydney | Australia |

*^Abbreviations: ISOQOL (International Society for Quality of Life Research); SIG (Special Interest Group); PROMs (Patient reported outcome measures); QUOKKA (QUality OF Life in Kids)^*
