# Supplementary material for: ‘How low can you go?’ Developers’ perspectives on involving young children in the development of patient reported outcome measures
Source: J Patient Rep Outcomes. 2025 Jul 15;9:91. doi: 10.1186/s41687-025-00924-y (PMC12263538; doi:10.1186/s41687-025-00924-y)
Supplement: Supplementary file 2 — Supplementary Material 2 [file 41687_2025_924_MOESM2_ESM.docx]

**Supplementary File 2: Online Survey**

**Article Title:** ‘How low can you go?’ Developers' perspectives on involving young children in the development of patient reported outcome measures

Start of Block: Introduction

**Introduction**

Thank you for agreeing to complete this survey. We are interested in understanding PROM developer's opinions about the involvement of children in qualitative aspects of PROM development (i.e., in concept elicitation and cognitive interviewing studies). The survey will take around 10-15 minutes to complete and you will be asked questions covering the following:

- Your background and demographics
- From what age children can participate in concept elicitation/cognitive interviewing
- Challenges associated with children's participation
- What information/evidence could demonstrate the feasibility of children's participation
- Your experience conducting concept elicitation/cognitive interviewing studies with or without children

All responses are anonymous.

End of Block: Introduction

Start of Block: Demographics

**SECTION 1**: **About You**

This survey is intended for PROM developers. We define "PROM developers" as having been listed as an author on **at least one** published PROM development paper (this CAN include the development of a descriptive system for a preference based measure (PBM), sometimes also known as a preference weighted measure (PWM)) **AND** having participated in **at least one** of the following activities as part of that project:

- Development of a conceptual model (e.g., concept elicitation)
- Item development
- Cognitive interviewing/debriefing to pre-test items
- Item reduction/selection
- Psychometric testing

Q1 By this definition, have you been listed as an author on a publication describing the development of a PROM?

- Yes (1)
- No (2)

Skip To: End of Block If By this definition, have you been listed as an author on a publication describing the development... = No

Q2 Which of the following have you been involved in, related to PROMs? *(Tick all that apply)*

- Development of a conceptual model (e.g., concept elicitation) (1)
- Item development (2)
- Cognitive interviewing/debriefing to pre-test items (3)
- Item reduction/selection (4)
- Psychometric testing (5)
- ⊗I have not been involved in any of the above (6)

Skip To: End of Block If Which of the following have you been involved in, related to PROMs? (Tick all that apply) = I have not been involved in any of the above

| Page Break |  |
| --- | --- |

Q3 How many years experience of developing PROMs do you have?

- 0 - 1 year (1)
- 2 - 4 years (2)
- 5 or more years (3)

Q4 In what setting are you primarily based?

- Academia (1)
- Industry (2)
- Other (please specify) (3) __________________________________________________

| 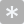 |
| --- |

Q5 Which country are you based in?

________________________________________________________________

| Page Break |  |
| --- | --- |

Q6 What is your age?

▼ Prefer not to say (1) ... 100 (87)

Q7 Would you describe yourself as a man, a woman, or in another way?

- Man (1)
- Woman (2)
- Another way (3)
- Prefer not to say (4)

Display this question:

If Would you describe yourself as a man, a woman, or in another way? = Another way

If you wish, you may specify how you describe yourself here: *Please do not include any identifying information in your response.*

________________________________________________________________

End of Block: Demographics

Start of Block: Not eligible

Display this question:

If Which of the following have you been involved in, related to PROMs? (Tick all that apply) = I have not been involved in any of the above

Thank you for your interest in completing this survey. Unfortunately, to be eligible to participate, you need to have been involved in at least one of the listed activities. Thank you again for your time.

Display this question:

If By this definition, have you been listed as an author on a publication describing the development... = No

Thank you for your interest in completing this survey. Unfortunately, to be eligible to participate, you need to have been listed as an author on at least one publication describing the development of a PROM. Thank you again for your time.

End of Block: Not eligible

Start of Block: Experience working with children

Q8 Are you a parent or guardian?

- Yes (1)
- No (2)

Display this question:

If Are you a parent or guardian? = Yes

Q9 Are you a parent or guardian to a child/children aged **11 years or younger**?

- Yes (1)
- No (2)

| Page Break |  |
| --- | --- |

Q10 Have you ever worked with children aged **11 years or younger** in a professional or voluntary capacity (e.g., researcher, teacher, healthcare professional, childcare worker, children's activities volunteer etc.)?

- Yes (1)
- No (2)

End of Block: Experience working with children

Start of Block: Definitions

**DEFINITIONS**:

This survey asks questions about the feasibility of children participating in concept elicitation and cognitive interviewing research. We define *participation*, *feasibility*, *concept elicitation*, and *cognitive interviewing* as follows:

**Participation**. We define "participation" as the child independently:

1. Engaging with the data collection task (e.g., listening to the interviewer, following task instructions)
2. Thinking about the interview questions/prompts (e.g., thinking about symptoms of a health condition)
3. Communicating their own views and ideas (e.g., verbally or non-verbally expressing thoughts, drawing pictures about a health condition if asked to by the interviewer)

Adults (in addition to the interviewer) may also be present, such as the child's parent or a healthcare professional known to the child. They may reasonably be expected to support the child provided they do not influence the child's answers. Examples of reasonable support may include:

- Supporting the child to read information
- Repeating or paraphrasing interview questions/prompts
- Gently re-directing the child back to the interview task

It would **NOT** include:

- Answering questions on behalf of the child
- Leading the child's thinking or communication of ideas

**Feasibility.** We define "feasibility" as it being possible to collect meaningful data from participants that is valuable to the aims of the research. With children, this may include the use of age-appropriate methods of data collection (e.g., shorter interview times, appropriate vocabulary for questions/prompts, potentially using props or creative activities). Feasibility does **NOT** require identical methods and processes to be used with children that are also used with adult participants.

**Concept elicitation research.** Qualitative research (e.g., interviews/focus groups) where representatives of the target population are asked about their experiences of the health condition. The information gathered is used to develop a conceptual framework and to help identify the health concepts to be included in the PROM and the generation of PROM content.

**Cognitive interviewing** (sometimes known as "cognitive debriefing"). Qualitative interviews conducted with representatives of the target population which aim to evaluate the content validity (comprehensiveness, comprehensibility, relevance) of the draft PROM. Typically the participant is asked to complete the draft PROM while also being asked direct verbal probes and/or to 'think aloud' such that the interviewer gains an insight into their thought processes. This information is used to evaluate the PROM's content validity.

End of Block: Definitions

Start of Block: Concept elicitation feasibility

**SECTION 2**: **Concept Elicitation**

The following questions ask about the feasibility of children participating in concept elicitation research. You can hover over key terms for a reminder of definitions throughout.

Q11 From what age do you think it is typically feasible for children to ***participate*** in *concept elicitation* research?

▼ 2 years (1) ... 18 years (17)

Q12 You stated that children younger than **${Q2/ChoiceGroup/SelectedChoices}** typically ***cannot participate*** in concept elicitation research Please indicate why below *(Tick all that apply)*

- They do not typically have the cognitive and/or linguistic skills needed (1)
- They typically are not reliable research participants (2)
- It is ethically inappropriate to involve them as research participants (3)
- They are not viewed as reliable research participants by others (e.g., peer reviewers, funding bodies) (4)
- It is too practically challenging to involve them (e.g., recruitment, obtaining ethical approval, finding space to conduct research) (5)
- There are not enough published examples of them having participated in concept elicitation research previously (6)
- There is not enough guidance (e.g., published guidelines) to support their involvement (7)
- Methods of data collection for this age are not sufficiently developed (8)
- Other (please specify) (9) __________________________________________________
- ⊗None of the above (10)

| Page Break |  |
| --- | --- |

Q13 You stated that children younger than **${Q2/ChoiceGroup/SelectedChoices}** typically ***cannnot*** ***participate*** in concept elicitation research. Which of the following, if any, would convince you otherwise? *(Tick all that apply)*

- Expert consensus study agreeing they can typically participate (1)
- More published examples of them having participated successfully (2)
- Empirical research that explores and demonstrates the feasibility of them participating (3)
- Specific guidance documents detailing how their participation can be enabled (4)
- Other (please specify) (5) __________________________________________________
- ⊗None. It would not be possible to demonstrate the feasibility of them participating in concept elicitation research (6)

End of Block: Concept elicitation feasibility

Start of Block: CE projects with children

The following questions are about developing PROMs for children, with self-report ***or*** proxy response options.

Q14 Have you ever been part* of a project/s developing a children's PROM where children (<18 years) have been included as participants in ***concept elicitation***? **this can include any role on the research project e.g., data analyst, data collector, principal investigator, part of the wider project team etc.*

- Yes, 1 project (1)
- Yes, 2 - 4 projects (2)
- Yes, 5 or more projects (3)
- No (4)

Display this question:

If Have you ever been part* of a project/s developing a children's PROM where children ( 18 years) h... = Yes, 1 project

Q15 Which ages of children participated in this project? *(Tick all that apply)*

- 1 year (1)
- 2 years (2)
- 3 years (3)
- 4 years (4)
- 5 years (5)
- 6 years (6)
- 7 years (7)
- 8 years (8)
- 9 years (9)
- 10 years (10)
- 11 years (11)
- 12 years (12)
- 13 years (13)
- 14 years (14)
- 15 years (15)
- 16 years (16)
- 17 years (17)

Display this question:

If Have you ever been part* of a project/s developing a children's PROM where children ( 18 years) h... = Yes, 2 - 4 projects

Or Have you ever been part* of a project/s developing a children's PROM where children ( 18 years) h... = Yes, 5 or more projects

Q16 Which ages of children participated across these projects? *(Tick all that apply)*

- 1 year (1)
- 2 years (2)
- 3 years (3)
- 4 years (4)
- 5 years (5)
- 6 years (6)
- 7 years (7)
- 8 years (8)
- 9 years (9)
- 10 years (10)
- 11 years (11)
- 12 years (12)
- 13 years (13)
- 14 years (14)
- 15 years (15)
- 16 years (16)
- 17 years (17)

Display this question:

If Have you ever been part* of a project/s developing a children's PROM where children ( 18 years) h... = Yes, 1 project

Q17 Thinking about this ***concept elicitation*** project, to what extent do you agree with the following?

|  | Agree (1) | Somewhat agree/Somewhat disagree (2) | Disagree (3) |
| --- | --- | --- | --- |
| Data collected from children was sufficient to answer research questions. (1) |  |  |  |
| Data collection methods (e.g., interview methods) could be adapted for the age/s of children participating. (2) |  |  |  |
| It was practically feasible (e.g., recruitment, finding time and space for data collection) to involve children. (3) |  |  |  |

Display this question:

If Have you ever been part* of a project/s developing a children's PROM where children ( 18 years) h... = Yes, 2 - 4 projects

Or Have you ever been part* of a project/s developing a children's PROM where children ( 18 years) h... = Yes, 5 or more projects

Q18 Thinking about these ***concept elicitation*** projects overall, to what extent do you agree with the following?

|  | Agree (1) | Somewhat agree/Somewhat disagree (2) | Disagree (3) |
| --- | --- | --- | --- |
| Data collected from children was typically sufficient to answer research questions. (1) |  |  |  |
| Data collection methods (e.g., interview methods) could typically be adapted for the age/s of children participating. (2) |  |  |  |
| It was typically practically feasible (e.g., recruitment, finding time and space for data collection) to involve children. (3) |  |  |  |

Display this question:

If Have you ever been part* of a project/s developing a children's PROM where children ( 18 years) h... = Yes, 1 project

Q19 Did you conduct interviews/lead a focus group (or similar) with children as part of this project?

- Yes (1)
- No (2)

Display this question:

If Have you ever been part* of a project/s developing a children's PROM where children ( 18 years) h... = Yes, 2 - 4 projects

Or Have you ever been part* of a project/s developing a children's PROM where children ( 18 years) h... = Yes, 5 or more projects

Q20 Have you ever conducted interviews/led a focus group (or similar) with children as part of any of these projects?

- Yes, once (1)
- Yes, 2 - 4 times (2)
- Yes, 5 or more times (3)
- No (4)

Display this question:

If Have you ever been part* of a project/s developing a children's PROM where children ( 18 years) h... = Yes, 1 project

Or Have you ever been part* of a project/s developing a children's PROM where children ( 18 years) h... = Yes, 2 - 4 projects

Or Have you ever been part* of a project/s developing a children's PROM where children ( 18 years) h... = Yes, 5 or more projects

The following questions are about developing children's PROMs specifically with ***self-report**** options for children. **self-report****CAN*** *include adults supporting children to read PROMs and physically enter/mark a response, provided the choice of answer comes directly from the child.*

Display this question:

If Have you ever been part* of a project/s developing a children's PROM where children ( 18 years) h... = Yes, 1 project

Or Have you ever been part* of a project/s developing a children's PROM where children ( 18 years) h... = Yes, 2 - 4 projects

Or Have you ever been part* of a project/s developing a children's PROM where children ( 18 years) h... = Yes, 5 or more projects

Q21 Have you ever been part of a project where younger children **could** have been included in concept elicitation but **weren't**, for any reason? *For example, the PROM was intended to have a self-report option for children aged 6-11 years, but only children aged 8-11 years participated in concept elicitation.*

- Yes, once (1)
- Yes, more than once (2)
- No (3)

Display this question:

If Have you ever been part of a project where younger children could have been included in concept e... = Yes, once

Or Have you ever been part of a project where younger children could have been included in concept e... = Yes, more than once

Q22 You stated that you have been part of a project/s where younger children **could** have been included in concept elicitation but **weren't**.   Please indicate why below. *(Tick all that apply)*

- Younger children were invited to participate but it was not possible to successfully recruit them (1)
- Younger children were not invited to participate because the concept elicitation task was considered to complex for them (2)
- Ethics approval was sought but not granted for the younger children to participate in concept elicitation (3)
- The concept elicitation task could not be adapted for the needs of the younger children (4)
- There was not enough guidance available (e.g., published guidelines, researcher training) to support the inclusion of the younger children (5)
- The research team did not have expertise required to include the younger children (6)
- Other (please specify) (7) __________________________________________________
- ⊗None of the above. (8)

End of Block: CE projects with children

Start of Block: Cognitive Interview Feasibility

**SECTION 3**: **Cognitive Interviewing**

The following questions ask about the feasibility of children participating in cognitive interviews. You can hover over key terms for a reminder of definitions throughout.

Q23 From what age do you think it is typically feasible for children to ***participate*** in *cognitive interviews*?

▼ 2 years (1) ... 18 years (17)

Q24 You stated that children younger than **${Q77/ChoiceGroup/SelectedChoices}** typically ***cannot*** ***participate*** in cognitive interviews. Please indicate why below *(Tick all that apply)*

- They do not typically have the cognitive and/or linguistic skills needed (1)
- They typically are not reliable research participants (2)
- It is ethically inappropriate to involve them as research participants (3)
- They are not viewed as reliable research participants by others (e.g., peer reviewers, funding bodies) (4)
- It is too practically challenging to involve them (e.g., recruitment, obtaining ethical approval, finding space to conduct research) (5)
- There are not enough published examples of them having participated in cognitive interviews previously (6)
- There is not enough guidance (e.g., published guidelines) to support their involvement (7)
- Methods of data collection for this age are not sufficiently developed (8)
- Other (please specify) (9) __________________________________________________
- ⊗None of the above (10)

Q25 You stated that children younger than **${Q77/ChoiceGroup/SelectedChoices}** typically ***cannot participate*** in cognitive interviews. Which of the following, if any, would convince you otherwise? *(Tick all that apply)*

- Expert consensus study agreeing they can typically participate (1)
- More published examples of them having participated successfully (2)
- Empirical research that explores and demonstrates the feasibility of them participating (3)
- Specific guidance documents detailing how their participation can be enabled (4)
- Other (please specify) (5) __________________________________________________
- ⊗None. It would not be possible to demonstrate the feasibility of them participating in cognitive interviews (6)

End of Block: Cognitive Interview Feasibility

Start of Block: CI projects with children

The following questions are about developing PROMs for children, with self-report ***or*** proxy response options.

Q26 Have you ever been part* of a project/s developing a children's PROM (self-report or proxy) where children (<18 years) have been included as participants in ***cognitive interviews***? **this can include any role on the research project e.g., data analyst, data collector, principal investigator, part of the wider project team etc.*

- Yes, 1 project (1)
- Yes, 2 - 4 projects (2)
- Yes, 5 or more projects (3)
- No (4)

Display this question:

If Have you ever been part* of a project/s developing a children's PROM (self-report or proxy) where... = Yes, 1 project

Q27 Which ages of children participated in this project? *(Tick all that apply)*

- 1 year (1)
- 2 years (2)
- 3 years (3)
- 4 years (4)
- 5 years (5)
- 6 years (6)
- 7 years (7)
- 8 years (8)
- 9 years (9)
- 10 years (10)
- 11 years (11)
- 12 years (12)
- 13 years (13)
- 14 years (14)
- 15 years (15)
- 16 years (16)
- 17 years (17)

Display this question:

If Have you ever been part* of a project/s developing a children's PROM (self-report or proxy) where... = Yes, 2 - 4 projects

Or Have you ever been part* of a project/s developing a children's PROM (self-report or proxy) where... = Yes, 5 or more projects

Q28 Which ages of children participated across these projects? *(Tick all that apply)*

- 1 year (1)
- 2 years (2)
- 3 years (3)
- 4 years (4)
- 5 years (5)
- 6 years (6)
- 7 years (7)
- 8 years (8)
- 9 years (9)
- 10 years (10)
- 11 years (11)
- 12 years (12)
- 13 years (13)
- 14 years (14)
- 15 years (15)
- 16 years (16)
- 17 years (17)

Display this question:

If Have you ever been part* of a project/s developing a children's PROM (self-report or proxy) where... = Yes, 1 project

Q29 Thinking about this ***cognitive interviewing*** project, to what extent do you agree with the following?

|  | Agree (1) | Somewhat agree/Somewhat disagree (2) | Disagree (3) |
| --- | --- | --- | --- |
| Data collected from children was sufficient to answer research questions. (1) |  |  |  |
| Cognitive interview methods could be adapted for the age/s of children participating. (2) |  |  |  |
| It was practically feasible (e.g., recruitment, finding time and space for data collection) to involve children. (3) |  |  |  |

Display this question:

If Have you ever been part* of a project/s developing a children's PROM (self-report or proxy) where... = Yes, 2 - 4 projects

Or Have you ever been part* of a project/s developing a children's PROM (self-report or proxy) where... = Yes, 5 or more projects

Q30 Thinking about these ***cognitive interview*** projects overall, to what extent do you agree with the following?

|  | Agree (1) | Somewhat agree/Somewhat disagree (2) | Disagree (3) |
| --- | --- | --- | --- |
| Data collected from children was typically sufficient to answer research questions. (1) |  |  |  |
| Cognitive interview methods could typically be adapted for the age/s of children participating. (2) |  |  |  |
| It was typically practically feasible (e.g., recruitment, finding time and space for data collection) to involve children. (3) |  |  |  |

Display this question:

If Have you ever been part* of a project/s developing a children's PROM (self-report or proxy) where... = Yes, 1 project

Q31 Did you conduct cognitive interviews with children as part of this project?

- Yes (1)
- No (2)

Display this question:

If Have you ever been part* of a project/s developing a children's PROM (self-report or proxy) where... = Yes, 2 - 4 projects

Or Have you ever been part* of a project/s developing a children's PROM (self-report or proxy) where... = Yes, 5 or more projects

Q32 Have you ever conducted cognitive interviews with children as part of any of these projects?

- Yes, once (1)
- Yes, 2 - 4 times (2)
- Yes, 5 or more times (3)
- No (4)

Display this question:

If Have you ever been part* of a project/s developing a children's PROM (self-report or proxy) where... = Yes, 1 project

Or Have you ever been part* of a project/s developing a children's PROM (self-report or proxy) where... = Yes, 2 - 4 projects

Or Have you ever been part* of a project/s developing a children's PROM (self-report or proxy) where... = Yes, 5 or more projects

The following questions are about developing children's PROMs specifically with ***self-report**** options for children. **self-report****CAN*** *include adults supporting children to read PROMs and physically enter/mark a response, provided the choice of answer comes directly from the child.*

Display this question:

If Have you ever been part* of a project/s developing a children's PROM (self-report or proxy) where... = Yes, 1 project

Or Have you ever been part* of a project/s developing a children's PROM (self-report or proxy) where... = Yes, 2 - 4 projects

Or Have you ever been part* of a project/s developing a children's PROM (self-report or proxy) where... = Yes, 5 or more projects

Q33 Have you ever been part of a project where younger children **could** have been included in cognitive interviews but **weren't**, for any reason? *For example, the PROM was intended to have a self-report option for children aged 6-11 years, but only children aged 8-11 years participated in concept elicitation.*

- Yes, once (1)
- Yes, more than once (2)
- No (3)

Display this question:

If Have you ever been part of a project where younger children could have been included in cognitive... = Yes, once

Or Have you ever been part of a project where younger children could have been included in cognitive... = Yes, more than once

Q34 You stated that you have been part of a project/s where younger children **could** have been included in cognitive interviews but **weren't**.   Please indicate why below. *(Tick all that apply)*

- Younger children were invited to participate but it was not possible to successfully recruit them (1)
- Younger children were not invited to participate because the concept elicitation task was considered to complex for them (2)
- Ethics approval was sought but not granted for the younger children to participate in concept elicitation (3)
- Cognitive interview methods could not be adapted for the needs of the younger children (4)
- There was not enough guidance available (e.g., published guidelines, researcher training) to support the inclusion of the younger children (5)
- The research team did not have expertise required to include the younger children (6)
- Other (please specify) (7) __________________________________________________
- ⊗None of the above. (8)

End of Block: CI projects with children

Start of Block: Other

Q35 If there is anything else you would like to tell us related to the feasibility of including children in concept elicitation or cognitive interviewing, please do so here: *Please do not include any identifying information in your response.*

________________________________________________________________

End of Block: Other
